# Differences in the Neanderthal *BRCA2* gene might be related to their distinctive cognitive profile

**DOI:** 10.1186/s41065-018-0076-2

**Published:** 2018-12-13

**Authors:** Antonio Benítez-Burraco

**Affiliations:** Department of Spanish, Linguistics, and Theory of Literature (Linguistics), University of Seville, Seville, Spain

**Keywords:** *BRCA2*, Neanderthal, Evolution, Cognition, Autism

## Abstract

The unique divergence of the *BRCA2* gene in Neanderthals compared to modern humans has been hypothesized to account for a differential susceptibility to cancer. However, the role of the gene in brain development and its connection with autism suggest that these differences might be (also) related to the more encapsulated nature of the Neanderthal cognition and their (inferred) autistic-like features.

## Main text

In their paper [1] Michalak and Kang report on three unique non-synonymous nucleotide substitutions in the Neanderthal breast cancer 2 (*BRCA2*) gene. According to the authors, these changes might be involved in a species-specific differential response to cancer, particularly, because one of the variants (position (2)) has been associated to two cancer types in present-day human populations. Although there are some descriptions of tumoral lesions in Neanderthals [2], there is no evidence of a differential susceptibility to cancer among them. Other reasons might account for the selection of these variants in Neanderthals. *BRCA2* is known to be required for normal neurogenesis [3] and mutations in the gene have been associated to intellectual disability and microcephaly [4]. Interestingly enough, two de novo missense mutations in *BRCA2* have been identified in individuals with autism spectrum disorder (ASD) [5]. The parallels between the Neanderthal and the ASD brains/minds are striking (reviewed in [6]). Candidate genes for ASD are overrepresented among the genes that have changed in the human lineage [7, 8]. Additionally, introgression events from archaic hominins have been claimed to account for aspects of the diversity of genes related to neurodevelopmental disorders, including ASD [9]. Finally, experimental and biochemical data suggest that BRCA2 interacts with several proteins involved in brain function and associated to cognitive disease, specifically, TP53 [10], PARP1 [11], and FLNA [12]. TP53 exhibits a human-specific variant not found in Neanderthals [13]. PARP1 regulates the dyslexia-susceptibility gene *DYX1C1* [14]. FLNA is involved in neuronal migration [15] and interacts with ITGB4 [16], a protein showing two fixed changes in humans compared to Neanderthals [17]. Overall, this evidence suggests that the reported changes in the Neanderthal *BRCA2* gene might have been (also) selected because of its impact on cognition, plausibly accounting for some of the differences between Neanderthal and human cognitive abilities.

## Abbreviations

ASD: autism spectrum disorder
BRCA2: breast cancer 2

## References

[1] Michalak P, Kang L. Unique divergence of the breast cancer 2 (BRCA2) gene in Neanderthals. Hereditas. 2018;155:34. 10.1186/s41065-018-0073-5 PMC6215347 30410429

[2] Colella G, Cappabianca S, Gerardi G, Mallegni F (2012). Homo neanderthalensis; first documented benign intraosseous tumor in maxillofacial skeleton. J Oral Maxillofac Surg.

[3] Frappart PO, Lee Y, Lamont J, McKinnon PJ. BRCA2 is required for neurogenesis and suppression of medulloblastoma. EMBO J. 2007;26(11):2732–42. 10.1038/sj.emboj.7601703 PMC1888666 17476307

[4] Rump P, Jazayeri O, van Dijk-Bos KK, Johansson LF, van Essen AJ, Verheij JB (2016). Whole-exome sequencing is a powerful approach for establishing the etiological diagnosis in patients with intellectual disability and microcephaly. BMC Med Genet.

[5] Neale BM, Kou Y, Liu L, Ma'ayan A, Samocha KE, Sabo A (2012). Patterns and rates of exonic de novo mutations in autism spectrum disorders. Nature.

[6] Benítez-Burraco A, Murphy E (2016). The oscillopatic nature of language deficits in autism: from genes to language evolution. Front Hum Neurosci.

[7] Polimanti R, Gelernter J (2017). Widespread signatures of positive selection in common risk alleles associated to autism spectrum disorder. PLoS Genet.

[8] Doan RN, Bae BI, Cubelos B, Chang C, Hossain AA, Al-Saad S (2016). Mutations in human accelerated regions disrupt cognition and social behavior. Cell.

[9] Mozzi A, Forni D, Cagliani R, Pozzoli U, Clerici M, Sironi M (2017). Distinct selective forces and Neanderthal introgression shaped genetic diversity at genes involved in neurodevelopmental disorders. Sci Rep.

[10] Marmorstein LY, Ouchi T, Aaronson SA. The BRCA2 gene product functionally interacts with p53 and RAD51. Proc Natl Acad Sci U S A. 1998;95(23):13869–74. 10.1073/pnas.95.23.13869 PMC24938 9811893

[11] Wang J, Bian C, Li J, Couch FJ, Wu K, Zhao RC. Poly(ADP-ribose) polymerase-1 down-regulates BRCA2 expression through the BRCA2 promoter. J Biol Chem. 2008;283(52):36249–56. 10.1074/jbc.M803693200 PMC2605989 18990703

[12] Yuan Y, Shen Z. Interaction with BRCA2 suggests a role for filamin-1 (hsFLNa) in DNA damage response. J Biol Chem. 2001;276(51):48318–24. 10.1074/jbc.M102557200 11602572

[13] Dd P, Paixão-Côrtes VR, Hainaut P, Bortolini MC, Ashton-Prolla P (2012). The TP53 fertility network. Genet Mol Biol.

[14] Tapia-Páez I, Tammimies K, Massinen S, Roy AL, Kere J (2008). The complex of TFII-I, PARP1, and SFPQ proteins regulates the DYX1C1 gene implicated in neuronal migration and dyslexia. FASEB J.

[15] Fox JW, Lamperti ED, Eksioglu YZ, Hong SE, Feng Y, Graham DA (1998). Mutations in filamin 1 prevent migration of cerebral cortical neurons in human periventricular heterotopia. Neuron.

[16] Travis MA, van der Flier A, Kammerer RA, Mould AP, Sonnenberg A, Humphries MJ (2004). Interaction of filamin a with the integrin beta 7 cytoplasmic domain: role of alternative splicing and phosphorylation. FEBS Lett.

[17] Pääbo S (2014). The human condition: a molecular approach. Cell.

